# Spectroscopic Analysis of Rare-Earth Silicide Structures on the Si(111) Surface

**DOI:** 10.3390/ma14154104

**Published:** 2021-07-23

**Authors:** Simone Sanna, Julian Plaickner, Kris Holtgrewe, Vincent M. Wettig, Eugen Speiser, Sandhya Chandola, Norbert Esser

**Affiliations:** 1 Institut für Theoretische Physik and Center for Materials Research (LaMa), Justus-Liebig-Universität Gießen, Heinrich-Buff-Ring 16, 35392 Gießen, Germany; kris.holtgrewe@theo.physik.uni-giessen.de (K.H.); Vincent.M.Wettig@physik.uni-giessen.de (V.M.W.); 2 Helmholtz-Zentrum Berlin für Materialen und Energie GmbH, Hahn-Meitner Platz 1, 14109 Berlin, Germany; julian.plaickner@helmholtz-berlin.de (J.P.); sandhya.chandola@helmholtz-berlin.de (S.C.); 3 Leibniz-Institut für Analytische Wissenschaften-ISAS-e.V., Schwarzschildstr. 8, 12489 Berlin, Germany; eugen.speiser@laytec.de (E.S.); norbert.esser@isas.de (N.E.); 4 Institut für Festkörperphysik, Technische Universität Berlin, Hardenbergstr. 36, 10623 Berlin, Germany

**Keywords:** surface science, Si(111), rare earth silicide, terbium silicide, Raman spectroscopy, RAS, density functional theory, DFT, thin films, 2D material

## Abstract

Two-dimensional rare-earth silicide layers deposited on silicon substrates have been intensively investigated in the last decade, as they can be exploited both as Ohmic contacts or as photodetectors, depending on the substrate doping. In this study, we characterize rare-earth silicide layers on the Si(111) surface by a spectroscopic analysis. In detail, we combine Raman and reflectance anisotropy spectroscopy (RAS) with first-principles calculations in the framework of the density functional theory. RAS suggests a weakly isotropic surface, and Raman spectroscopy reveals the presence of surface localized phonons. Atomistic calculations allow to assign the detected Raman peaks to phonon modes localized at the silicide layer. The good agreement between the calculations and the measurements provides a strong argument for the employed structural model.

## 1. Introduction

Rare earth (RE) deposition on Si substrates followed by thermal annealing leads to the formation of various structures consisting of rare-earth silicides of different composition. The amount of deposited rare earths, the substrate orientation, and the annealing process can be exploited to obtain structures of different dimensionality (from one dimensional (1D) to three-dimensional (3D)), periodicity, and morphology.

Quasi-1D metallic nanowires of different rare-earth silicides [1,2,3,4,5,6] grow on Si(001) substrates and vicinal surfaces [7,8,9,10,11,12,13,14,15]. Due to their highly anisotropic growth, the wires are interesting model systems for the realization of 1D physics [16,17,18,19,20] or as building blocks for nanoelectronics applications [21,22,23,24,25].

The 2D films of rare-earth silicides can be epitaxially grown on the Si(111). Most trivalent rare-earth elements lead to surface reconstructions of related morphology and electronic properties [26,27,28,29,30,31,32,33,34]. The silicide layers have been exploited both as Ohmic contacts (due to the low Schottky-barrier heights) on *n*-type Si substrates or as photodetectors on *p*-type Si substrates [35,36].

Among the known rare-earth induced reconstructions of the Si(111) surface, a metallic phase consisting of a regular, stoichiometric ${\mathrm{RESi}}_{2}$ silicide monolayer (ML) of (1 × 1) periodicity, and a further metallic phase consisting of a regular ${\mathrm{RE}}_{3}$${\mathrm{Si}}_{5}$ silicide bilayer of ($\sqrt{3}\times \sqrt{3}$) periodicity are the most intensively investigated structures.

Although different experimental studies have been dedicated to the characterization of the silicide layers, their lattice dynamics is completely unknown. In this manuscript, we investigate experimentally (on the example of terbium silicide) and theoretically (for all lanthanides) the vibrational properties of silicide layers of different height. Surface-localized phonon modes can be observed at the terbium silicide surface layer, which do not substantially depend on the system temperature. Moreover, optical absorption features are identified in reflectance anisotropy spectroscopy (RAS), which can be related to interband transitions within the Tb-Si surface electronic band structure.

Corresponding atomistic calculations allow to assign the detected Raman peaks to phonon modes in the (1 × 1)-periodic monolayer structure, which are localized in the silicide. A roughly linear dependence of the Raman shifts on the atomic number of the considered rare earth is predicted, which correlates with the bond lengths of the different silicides. Structure specific phonon modes are found for the silicide monolayer of (1 × 1) periodicity and for the silicide bilayer of ($\sqrt{3}\times \sqrt{3}$) periodicity, which, in principle, allow for a structural determination on the basis of the Raman spectra.

The good agreement between the calculations and the measurements strongly supports the employed structural model.

## 2. Materials and Methods

### 2.1. Sample Preparation

Vicinal Si(111) substrates with a miscut angle of 4 ± 1° degree were cut from commercial *n*-type Si(111) wafers and cleaned in an ultra-high vacuum (UHV) by degassing for 12 h at 600 °C. Then, repeated flash annealing up to 1200 °C was applied followed by slow cooling down from 850 °C at a rate of about 1 °C/s to obtain a well-ordered clean (7 × 7) surface. Spot splitting of the integer spots were observed by low energy electron diffraction (LEED, not shown) due to the presence of steps resulting from the miscut angle. Annealing temperatures were measured by an infrared pyrometer. The Tb silicide structures were grown in situ by depositing Tb onto the clean Si(111)-(7 × 7) surface held at room temperature followed by annealing at 520 °C for 2 min to form the silicide structures. In this work, the Tb coverage is given in monolayers (ML), with 1 ML corresponding to the surface atom density at the unreconstructed Si(111) surface (7.8 × $10^{14}$ atoms/${\mathrm{cm}}^{2}$). The base pressure was better than 1 × $10^{-10}$ mbar and did not exceed 5 × $10^{-10}$ mbar during preparation, ruling out any form of contamination. The deposited Tb amount was determined by identifying the parameters for the different growth regimes, namely the submonolayer, monolayer, and multilayer regimes known from previous publications [28].

For submonolayer Tb coverages (about 0.4 ML Tb annealed at 550 °C), a ($2\sqrt{3}\times 2\sqrt{3}$) R30° superstructure is observed with LEED, in agreement with available data [28]. In the monolayer regime, (about 1 ML Tb, annealed at 550 °C) the ${\mathrm{TbSi}}_{2}$ monolayer with (1 × 1) periodicity described in the following sections is formed, while a strong ($\sqrt{3}\times \sqrt{3}$) R30° LEED pattern is observed for the multilayer regime (coverage exceeding 1 ML Tb), which is again in agreement with the literature [28]. We remark that between the monolayer and multilayer regime, Tb silicide islands of different morphology and periodicity start to grow, which coalesce to form closed layers after thermal treatment. These structures correspond either to the two-dimensional hexagonal ${\mathrm{TbSi}}_{2}$ monolayer with (1 × 1) periodicity or to the higher hexagonal ${\mathrm{Tb}}_{3}$${\mathrm{Si}}_{5}$ multilayer structures with ($\sqrt{3}\times \sqrt{3}$) periodicity, which are also described in the following. This regime is called the monolayer-to-multilayer regime [28].

In our experimental investigation, we employ Tb as a representative trivalent rare-earth. Tb silicide phases belong to the group of silicide structures of the trivalent rare-earth metals, which have very similar structural, chemical, and electronic properties [26]. They are described by the same structural models, based on the defect-free, hexagonal rare-earth disilicides. This notwithstanding, slightly differing procedures might be necessary to crystallize silicides of different rare earths. Although the growth of Tb on Si(111) displays very similar characteristics as observed for other trivalent rare-earth metals such as Gd, Dy, or Er, which all feature the previously described growth regimes [28,37,38], some of the light rare earths might present peculiarities in their growth process. The discussion of the growth process of the different lanthanides is beyond the scope of this investigation, though.

### 2.2. Experimental Setup

Raman measurements were obtained using a Dilor triple spectrometer and a Si-based high-efficiency CCD detector. The excitation wavelength of 458 nm (2.70 eV photon energy) was generated by an ${\mathrm{Ar}}^{+}$ ion laser operated at a power of approximately 400 mW. The Raman spectra were calibrated using 9 different ${\mathrm{Ar}}^{+}$ laser plasma lines. The spectral resolution was obtained from the Gauss width of the plasma lines, and is equal to 3.5 ${\mathrm{cm}}^{-1}$. The measured Raman spectra contain bulk and surface contributions that were separated from each other according to the procedure described in Appendix A.

**Figure 1:**
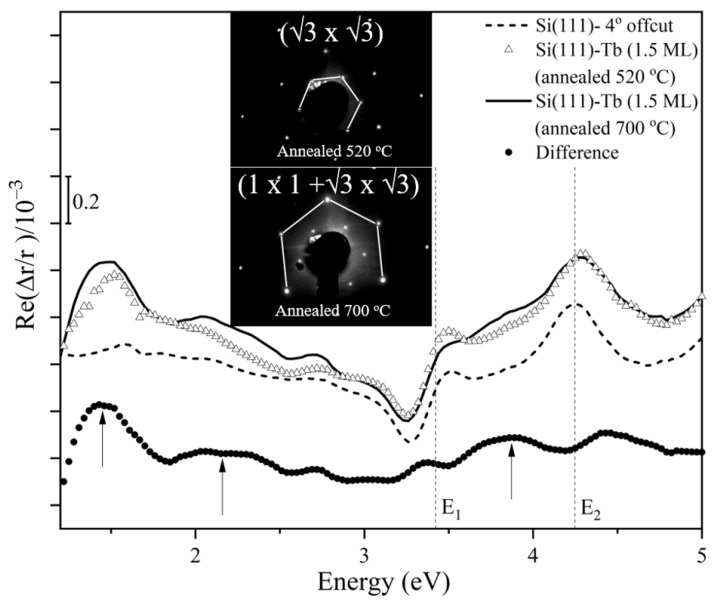
Reflectance anisotropy spectrum obtained before and after deposition of 1.5 ML Tb on the 4°-offcut Si(111) substrate and annealing at different temperatures. The upper (lower) inset shows the corresponding LEED pattern after annealing at 520 °C (700 °C).

Figure 1 shows a RAS spectrum obtained with 1.5 ML Tb on the Si(111) substrate. The arrows indicate relevant optical transitions at 1.4, 2.2, and 3.8 eV. According to the electronic band structure published in previous works [8,26], the optical absorption seems to be related to electronic transitions within the surface electronic band structure, which becomes anisotropic due to presence of the step edges on the vicinal Si substrate.

The LEED pattern in the upper inset of Figure 1 shows a ($\sqrt{3}\times \sqrt{3}$) reconstruction. However, no modes at all were detected in Raman measurements for this surface. Raman signatures could be only detected after a further annealing at a higher temperature of 700 °C. The LEED analysis (lower inset of Figure 1) showed that the bulk (1 × 1) spots became much stronger than those of the surface annealed at 520 °C. The ($\sqrt{3}\times \sqrt{3}$) spots are still visible, though less sharp and with a much higher background. This indicates the formation of a broad, surface-covering (1 × 1) monolayer, which contains smaller ($\sqrt{3}\times \sqrt{3}$) islands. This is in agreement with the previously mentioned coalescence of silicide islands into homogeneous layers in the monolayer-to-multilayer regime [28].

**Figure 2:**
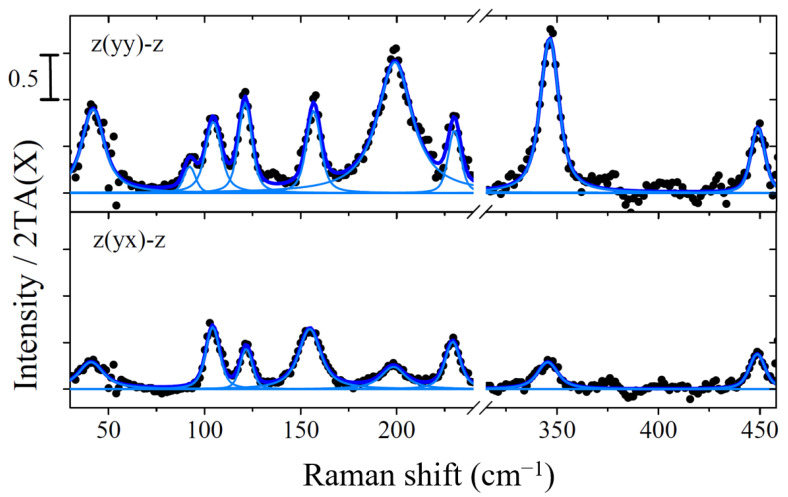
Surface Raman spectra of the terbium silicide layer grown on 4°-offcut Si(111) substrate for parallel z(yy)-z and crossed z(yx)-z polarization configurations. The spectra are fitted with Voigt profiles with a fixed Gauss width equal to the spectral resolution of 3.5 ${\mathrm{cm}}^{-1}$.

**Figure A2:**
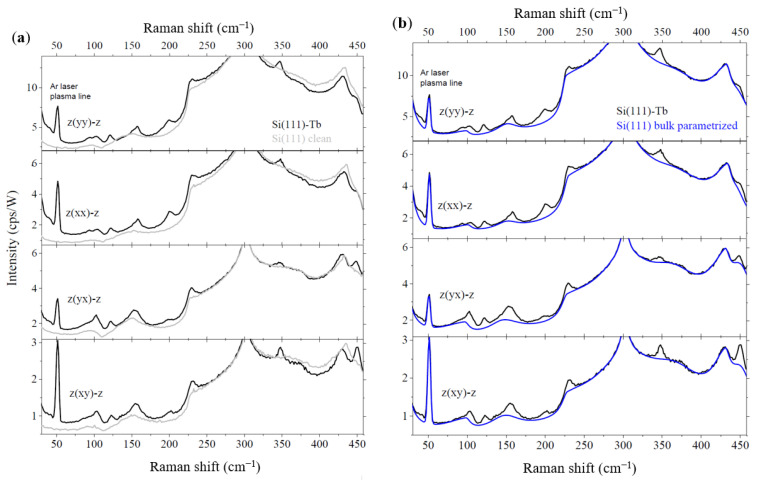
Measured Raman spectra of terbium silicide layer grown on 4°-offcut Si(111) substrate (black) are compared with (**a**) the measured spectra of the clean Si(111) surface (grey) and (**b**) the parametrized spectra of bulk Si(111) (blue). The Ar laser plasma line at 50 ${\mathrm{cm}}^{-1}$, visible due to misalignment of the bandpass filter, was included in the parametrization of the bulk.

We would like to note that the surface Raman spectra, as shown in Figure 2 or Figure A2 in Appendix A, are always reproducible after the annealing at 700 °C, while at lower annealing temperatures no surface modes are visible at all. This may be related to the complex stoichiometry of the Tb/Si surface, which consists of a mix of different phases after annealing at low temperatures, and develops into a larger fraction of (1 × 1) islands only after the high-temperature annealing [28,39]. Moreover, the surface morphology may contribute to an enhancement of the surface Raman signal, for instance due to momentum conservation relaxation or by local field enhancement effects. Finally, we note that the surface Raman signal was sensitively dependent on small contamination by residual gas, since the surface Raman signal of fresh preparations diminished rapidly within a few hours in UHV. This supports the surface origin of the recorded Raman signal.

### 2.3. Computational Details

The spectroscopic signatures of the ${\mathrm{TbSi}}_{2}$ monolayer on the Si(111) surface are modeled within density functional theory (DFT). The calculations are performed in the framework of the generalized gradient approximation [40] (GGA) in the PBEsol formulation [41,42] as implemented in the Vienna ab initio simulation package (VASP) [43,44].

Projector augmented wave [45,46] (PAW) potentials with projectors up to l=1 for H, l=2 for Si and l=3 for Tb and the other rare earths have been used. As no other valence state than ${\mathrm{RE}}^{3+}$ has been observed for Tb ions in the silicide structures, we constrain the Tb valence state treating n−1 *f*-electrons as core states. This approach, commonly referred to as frozen-core method, allows for a proper treatment of the lanthanides within DFT [47,48,49]. We have verified in a previous work [1] that keeping the *f* electrons frozen in the atomic core plays a negligible role in the structural properties of stochiometric bulk hexagonal rare earth silicides, leading only to variations in the lattice parameters smaller than 0.01 Å.

To ensure numerical convergence, the electronic wave functions are expanded into plane waves up to an energy cutoff of 400 eV, while the Brillouin zone is sampled by a 20×20×1Γ-centered Monkhorst-Pack mesh [50] in the case of the monolayer structure with (1 × 1) periodicity and by a 12×12×1Γ-centered mesh in the case of the bilayer structure with ($\sqrt{3}\times \sqrt{3}$) periodicity.

Planar ${\mathrm{TbSi}}_{2}$ silicide layers on the Si(111) are modeled with slabs consisting of 10 Si layers stacked along the (111) crystallographic direction (modeling the substrate), the silicide monolayer, and a vacuum region of at least 20 Å. The dangling bonds at the bottom face of the slabs are saturated by hydrogen atoms. Structural optimization is performed until the residual Hellmann–Feynman forces [51] are lower than 0.001 eV/Å. In this procedure, the silicide layer and 6 Si bilayers are free to relax, while 4 Si layers and the hydrogen atoms are kept fixed at the bulk positions in order to model the substrate.

## 3. Results

### 3.1. Vibrational Modes of the Tb-Layer on 4°-Offcut Si(111)

Figure 2 shows the surface Raman spectra of the terbium silicide layer on 4°-offcut Si(111) substrates for different polarization configurations. The spectra are fitted with Voigt profiles with a fixed Gauss width equal to the spectral resolution of 3.5 ${\mathrm{cm}}^{-1}$. Measured Raman frequencies and mode symmetry according to polarization selection rules are indicated in Table 1.

Within the threefold symmetry of the ideal hexagonal Si(111) surface, phonon modes of *A* and *E* symmetry are expected. Due to the Raman selection rules, *A* modes are observable in parallel polarization (the symmetry equivalent xx or yy configurations), while *E* modes are detectable within crossed polarization (the symmetry equivalent xy and yx configurations). Due to the symmetry reduction by atomic steps (4° offcut) and the possibly inhomogeneous surface morphology, the symmetry properties are not strictly mirrored in Raman experiments, but according changes in scattering intensity are still observable. Indeed, the Raman spectra measured in crossed and parallel polarization configuration show some differences. The bands at 41.5 ${\mathrm{cm}}^{-1}$, 198.8 ${\mathrm{cm}}^{-1}$ and 346.4 ${\mathrm{cm}}^{-1}$ exhibit much higher intensity in parallel polarization, while the bands at 104.3 ${\mathrm{cm}}^{-1}$ and 156.8 ${\mathrm{cm}}^{-1}$ are of comparable intensity in both scattering configurations.

On the basis of the relative Raman intensity measured in parallel and crossed polarization, we tentatively assign the mode symmetry as shown in the third column of Table 1. The close agreement of spectra recorded in xx and yy configuration, as well as in xy and yx configuration (shown in Figure A2 in the Appendix A), demonstrates the high reproducibility of the difference spectra we employ to characterize the silicide layers.

The Raman modes observed in the surface spectrum overlap, at least partially, with the bulk phonon branches of Si. As shown previously on different Si(hhk)-Au systems, surfaces resonances may arise by coupling to bulk phonon modes close to the Brillouin zone boundaries, activated by the surface modification [52]. Therefore, part of the observed modes will have predominant bulk character, while others will be mostly surface localized. This issue will be addressed in detail by the *ab initio* calculations described in the following.

**Figure 3:**
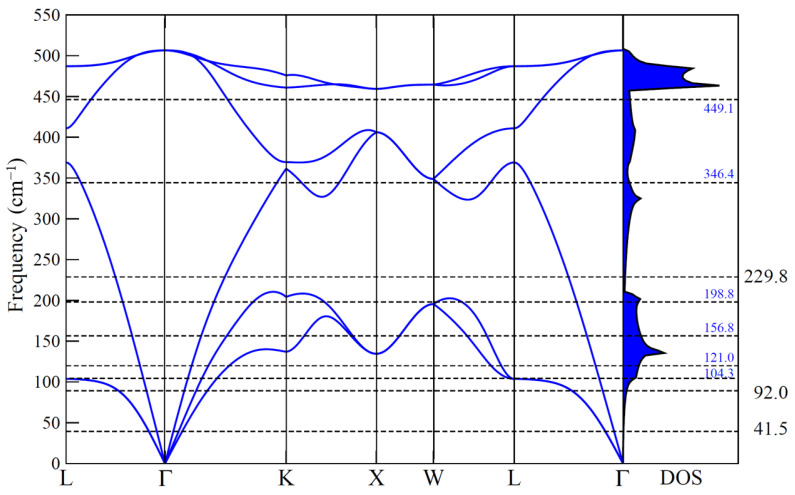
DFT-PBEsol calculated phonon dispersion (left hand side) and phonon density of states (right hand side) of bulk Si. The measured Raman frequencies (in parallel polarization) are represented as dashed horizontal lines.

To discriminate whether the measured Raman signatures are related to true surface localized phonon modes or rather to surface-activated bulk resonances, we compare them with the phonon dispersion of bulk Si, which we have calculated within DFT-PBEsol. Figure 3 shows the calculated dispersion and the corresponding density of states (DOS), in which we overlay the measured Raman signatures as horizontal dashed lines.

Although the modes measured at 41.5 ${\mathrm{cm}}^{-1}$, 92.0 ${\mathrm{cm}}^{-1}$, and 229.8 ${\mathrm{cm}}^{-1}$ are in non-resonant regions of low phonon DOS and can be safely regarded as surface modes, the situation is different for the other Raman peaks. The signatures at 104.3 ${\mathrm{cm}}^{-1}$, 121.0 ${\mathrm{cm}}^{-1}$, 156.8 ${\mathrm{cm}}^{-1}$, 198.8 ${\mathrm{cm}}^{-1}$, and 346.4 ${\mathrm{cm}}^{-1}$, are close to critical points of the phonon dispersion and might be either surface activated resonances or the overlap of surface localized modes and bulk resonances. Surface-activated bulk resonances were already reported for Au superstructures on different vicinal Si(111) substrates at very similar frequencies and were attributed to the altered boundary conditions by the metallic superstructures [52]. Most probably, the signature measured at 449.1 ${\mathrm{cm}}^{-1}$ is related to the optical phonon, which becomes very flat at the K point of the Brillouin zone, resulting in a very high phonon DOS just above 450 ${\mathrm{cm}}^{-1}$.

The modes which cannot be assigned to bulk resonances must be obviously related to the silicide structures formed on the Si(111) substrate. To investigate the origin of all observed modes, we model the vibrational properties the silicide monolayer and the silicide bilayer, which are the only structures which might be formed under the present experimental conditions.

### 3.2. The Monolayer ${RESi}_{2}$ Reconstruction

Density functional theory calculations have been performed to interpret the experimental results, to verify the tentative assignment of mode symmetry and surface localization shown in Table 1, and to assign a displacement pattern to the measured Raman signatures. We remark, at this point, that we employ flat silicide structures to model experiments performed on terraced, vicinal surfaces. Although models for silicide layers on certain vicinal Si surfaces are available, they basically represent the silicide structure known for flat substrates, with a step edge strongly dependent on the offcut angle. As an example rare-earth silicides grown on the Si(557)-surface [8], have been studied, which are obtained with a cutoff angle of +9.5° and very broad terraces larger than 18 Å. Thus, the even broader terraces resulting from the experimental offcut angle of +4° can be efficiently modeled, in first approximation, by the much simpler flat structures.

As the phonon frequencies calculated for the different rare earths depend on the system symmetry, as well as on the interatomic bond lengths, we start with a short description of the employed structural model.

#### 3.2.1. Structural Properties

**Figure 4:**
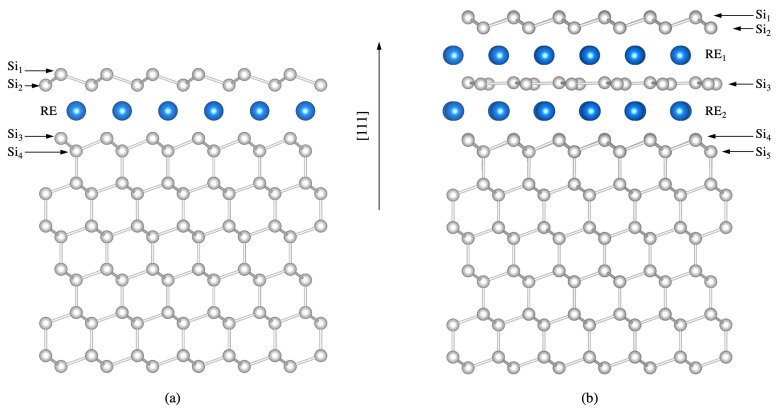
(**a**) Structural model of the rare-earth silicide monolayer with (1 × 1) periodicity on the Si(111) surface. (**b**) Rare-earth silicide bilayer with ($\sqrt{3}\times \sqrt{3}$) periodicity on the Si(111) surface. Si atoms are white, rare-earth atoms are blue. The topmost Si atomic layers are labeled by ${\mathrm{Si}}_{1}$-${\mathrm{Si}}_{5}$.

Previous studies showed that the deposition of rare earth silicide monolayers on the Si(111) surface results in the formation of stoichiometric films with (1 × 1) periodicity [26]. The commonly accepted model, firstly proposed for ${\mathrm{ErSi}}_{2}$-Si(111) [53] and then extended to other trivalent rare earths [26,54,55,56], is shown in Figure 4. It features a rare-earth silicide monolayer with the a hexagonal structure of the bulk silicide on top of a buckled, truncated-bulk Si(111)-surface. Interestingly, the orientations of the buckled Si top layer of the silicide and that of the Si(111) substrate buckled top layer are opposite. We accept this structural model for all trivalent rare earths and employ it for the calculations of the vibrational properties.

The distances between the atomic layers within this model depends on the considered rare earth. Table 2 shows that with growing atomic number the silicide layer height (as quantified, e.g., by the ${\mathrm{Si}}_{2}$-${\mathrm{Si}}_{3}$ vertical distance) decreases, while the corrugation of the buckled Si top layer of the silicide (${\mathrm{Si}}_{1}$-${\mathrm{Si}}_{2}$) and of the substrate termination (${\mathrm{Si}}_{3}$-${\mathrm{Si}}_{4}$) slightly increases. This nicely mirrors the lanthanide contraction, i.e., the contraction of the rare-earth ionic radii with increasing atomic number. The calculated values are in good agreement with previous calculations (where available) [26] and confirm that the interatomic distances in the silicide monolayer are close to those of the corresponding silicide bulk.

#### 3.2.2. Vibrational Properties of ${\mathrm{TbSi}}_{2}$

The phonon modes of the ${\mathrm{RESi}}_{2}$ monolayer structure at the Si(111) surface have been calculated according to the structural model presented in the previous section within the frozen-phonon method. Doubly degenerate phonon modes of *E* symmetry and non-degenerate *A* modes preserving the three-fold rotational symmetry are predicted. Their Raman intensity is calculated with the approach described in [57,58]. The modes predicted for the ${\mathrm{TbSi}}_{2}$ monolayer structure of (1 × 1) periodicity are compiled in Table 3.

We describe the phonons as *surface localized*, if their displacement pattern is localized by more than 35% within the layers ${\mathrm{Si}}_{1}$-${\mathrm{Si}}_{5}$. Although this is an arbitrarily chosen threshold, it allows to roughly discriminate between surface-localized modes and non-localized modes, which we assign to surface resonances of bulk modes. All Raman active modes which are surface localized can be associated with experimentally detected modes, the only exception being the mode predicted at 166.8 ${\mathrm{cm}}^{-1}$. This mode of *A* symmetry is a rigid vertical displacement of the whole ${\mathrm{Si}}_{1}$-${\mathrm{Si}}_{2}$ silicide termination, which is probably experimentally quenched by the less homogeneous surface relaxation due to the Si terraces. Among the experimentally detected modes that might be related to critical points of the bulk phonon dispersion (shown in Figure 3), the modes at 104.3 ${\mathrm{cm}}^{-1}$, 346.4 ${\mathrm{cm}}^{-1}$ and 449.1 ${\mathrm{cm}}^{-1}$ can be only assigned to bulk resonances, as the calculations do not predict surface localized modes of similar frequency. Instead the modes measured at 121.0 ${\mathrm{cm}}^{-1}$, 156.8 ${\mathrm{cm}}^{-1}$, and 229.8 ${\mathrm{cm}}^{-1}$ are close to calculated surface localized modes, wherefore they cannot clearly be assigned to bulk resonances or surface-localized phonon modes.

**Figure 5:**
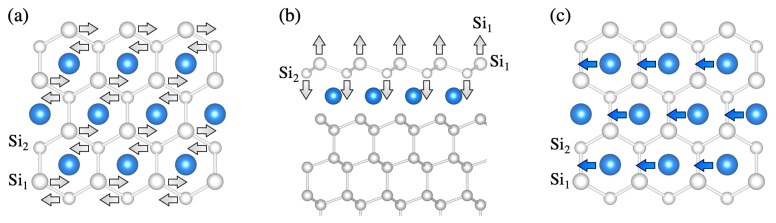
Displacement patterns of selected surface localized modes of ${\mathrm{TbSi}}_{2}$-silicide monolayers with (1 × 1)-periodicity calculated within DFT-PBEsol at (**a**) 467.6 ${\mathrm{cm}}^{-1}$, (**b**) 244.7 ${\mathrm{cm}}^{-1}$, and (**c**) 151.6 ${\mathrm{cm}}^{-1}$.

The modes with the highest surface localization are a lateral shearing of the ${\mathrm{Si}}_{1}$-${\mathrm{Si}}_{2}$ bilayer (467.6 ${\mathrm{cm}}^{-1}$, 92.7%, Figure 5a), a similar shearing of the ${\mathrm{Si}}_{3}$-${\mathrm{Si}}_{4}$ bilayer (414.3 ${\mathrm{cm}}^{-1}$, 97.0%), a vertical shearing of the ${\mathrm{Si}}_{1}$-${\mathrm{Si}}_{2}$ bilayer (244.7 ${\mathrm{cm}}^{-1}$, 82.5%, Figure 5b), a rigid lateral displacement of the Tb layer (151.6 ${\mathrm{cm}}^{-1}$, 97.7%, Figure 5c) and a rigid lateral translation of the ${\mathrm{Si}}_{3}$-${\mathrm{Si}}_{4}$ bilayer (117.8 ${\mathrm{cm}}^{-1}$, 84.9%).

As all of the experimentally detected Raman signatures can be assigned on the basis of their symmetry, frequency, and assumed surface localization to modes predicted with the silicide monolayer structure, we conclude that the silicide monolayer structure satisfactorily describes the measured terbium silicide Raman spectra. For the sake of completeness, we extend our calculations to the other rare earth silicides.

#### 3.2.3. Vibrational Properties of Rare Earth Silicides

The phonon modes calculated for the ${\mathrm{TbSi}}_{2}$ monolayer are the basis for understanding the vibrational properties of the monolayer structures of the other rare earths. The corresponding phonon frequencies have been compiled in Table A1 in Appendix B. Very similar frequencies are predicted for most rare-earth silicides.

**Figure 6:**
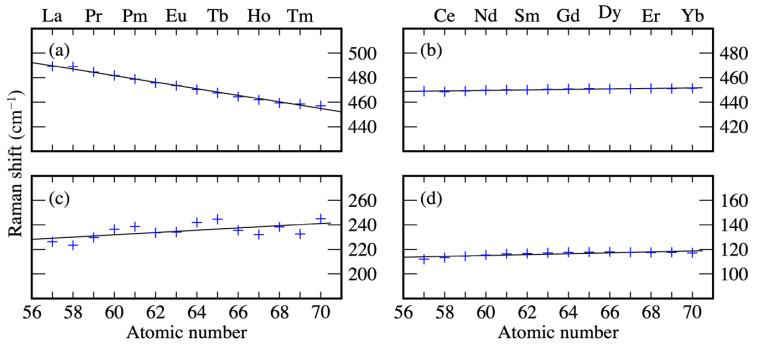
Raman shifts of selected surface localized modes of ${\mathrm{RESi}}_{2}$-Silicide monolayers with (1 × 1)-periodicity as a function of the rare earth calculated within DFT-PBEsol. Modes in (**a**,**c**) are a lateral and a vertical shearing of the ${\mathrm{Si}}_{1}$-${\mathrm{Si}}_{2}$ bilayer, respectively. Modes in (**b**,**d**) are vertical displacement of the Si atoms below the silicide monolayer and a lateral displacement of the substrate top bilayer, respectively.

Only two modes strongly localized at the silicide layer display a consistent dependence on the rare earth and shift by more than 20 ${\mathrm{cm}}^{-1}$ from La to Yb. The first is the lateral shearing of the ${\mathrm{Si}}_{1}$ and ${\mathrm{Si}}_{2}$ atoms of the topmost Si bilayer shown in Figure 5a. This *E* mode shifts from 489.2 ${\mathrm{cm}}^{-1}$ for ${\mathrm{LaSi}}_{2}$ down to 457.1 ${\mathrm{cm}}^{-1}$ for ${\mathrm{YbSi}}_{2}$, as represented in Figure 6a. The second mode is a vertical shearing of the same atoms displayed in Figure 5b. This *A* mode shifts from 226.2 ${\mathrm{cm}}^{-1}$ for ${\mathrm{LaSi}}_{2}$ up to 245.0 ${\mathrm{cm}}^{-1}$ for ${\mathrm{YbSi}}_{2}$, as represented in Figure 6c.

The predicted frequency dependence correlates with the structural buckling of the silicide. As the buckling of the topmost layer (${\mathrm{Si}}_{1}$-${\mathrm{Si}}_{2}$ vertical distance, shown in Table 2) grows with the atomic number, modes further enhancing the buckling become more energetic. For this reason, the mode shown in Figure 6c shifts to higher frequencies with growing atomic number.

On the contrary, a lateral shearing of the ${\mathrm{Si}}_{1}$-${\mathrm{Si}}_{2}$ atomic layers shows the opposite trend. In structures with low buckling, in which ${\mathrm{Si}}_{1}$ and ${\mathrm{Si}}_{2}$ are closer (light rare earths), the shear mode is hindered and, therefore, hard. For structures with more pronounced buckling, where ${\mathrm{Si}}_{1}$ and ${\mathrm{Si}}_{2}$ layers are further apart (heavy rare earths), the mode becomes softer.

Interestingly, the frequency shift of these two modes is higher than the frequency shift of other phonon modes directly involving the rare earths, suggesting that the bond strength influences the phonon frequency to a larger extent than the atomic mass.

All other modes show a moderate dependence or no dependence at all on the rare earth, as shown exemplarily for an *A* mode at about 450 ${\mathrm{cm}}^{-1}$ (vertical displacement of the Si atoms below the silicide, Figure 6b) and for an *E* mode at about 115 ${\mathrm{cm}}^{-1}$ (lateral displacement of the substrate top bilayer, Figure 6d).

### 3.3. The Bilayer ${RE}_{3}$${Si}_{5}$ Reconstruction

Although the modes calculated for the silicide monolayer satisfactorily explain the measured spectra, we calculate the vibrational properties of silicide bilayers. Indeed, the differences between the phonons in the monolayer and bilayer structure might lead to the identification of the silicide thickness.

#### 3.3.1. Structural Properties

Depositing more than one rare-earth monolayer on the Si(111) surface leads to the growth of multilayer structures, whose height depends on the rare-earth coverage [26,32,38,59,60]. In order to model the vibrational properties of the structures possibly obtained with the experimentally considered coverage, we limit ourselves to the simulation of bilayer structures. According to the commonly accepted model, rare-earth silicide bilayers at the Si(111) surface form structures with ($\sqrt{3}\times \sqrt{3}$) periodicity of ${\mathrm{RE}}_{3}$${\mathrm{Si}}_{5}$ stoichiometry rotated by 30° with respect to the substrate. This model (shown in Figure 4) features two rare-earth planes (${\mathrm{RE}}_{1}$ and ${\mathrm{RE}}_{2}$) separated by a flat, silicene-like Si layer (${\mathrm{Si}}_{3}$ in the picture). The silicene-like layer incorporates a Si vacancy, which is supposed to release the compressive strain present in the planar silicene-like film. The exact position of the Si vacancy has been debated in the past [61,62,63]. As we have previously modeled [26] that a Si vacancy below the atoms of the ${\mathrm{Si}}_{1}$ layer is energetically favorable by about 50 meV per surface unit cell for Y, Tb, Dy, and Er, we adopt this configuration for all the rare earths (We remark, however, that the energy difference between structures featuring the Si vacancy below the ${\mathrm{Si}}_{1}$ layer and below the ${\mathrm{Si}}_{2}$ layer is much lower than the thermal energy available during the film growth. As an homogeneous vacancy distribution is required to release strain, domains with vacancies below the ${\mathrm{Si}}_{2}$ atomic layer might freeze upon cooling). The silicide top layer is a buckled Si-bilayer, which, in contrast to the (1 × 1) monolayer structure, has the same orientation as the substrate. As further information concerning the silicides of the light rare earths are not available, we adopt the described structural model for all the lanthanides.

Table 4 shows that, similarly to the behavior of the silicide monolayer, the height of the silicide bilayer (e.g., ${\mathrm{Si}}_{2}$-${\mathrm{Si}}_{4}$ distance) decreases with the atomic number of the considered rare earth, while the buckling of both the topmost Si-bilayer (${\mathrm{Si}}_{1}$-${\mathrm{Si}}_{2}$), as well as that of the Si substrate ${\mathrm{Si}}_{4}$-${\mathrm{Si}}_{5}$ grows with the rare-earth atomic-number. The theoretical results are in very good agreement with previous calculations (where available) [26].

#### 3.3.2. Vibrational Properties

The modes predicted for the ${\mathrm{RESi}}_{2}$ bilayer structure of ($\sqrt{3}\times \sqrt{3}$) periodicity are compiled in Table A2 in Appendix B. Among these, some of the surface modes are common to the monolayer and bilayer structure. As an example, the lateral and the vertical shearing movement of the ${\mathrm{Si}}_{1}$-${\mathrm{Si}}_{2}$ layer represented in Figure 5a,b, occur both in the silicide monolayer and in the silicide bilayer as their termination is rather similar. Even the mode frequency is similar in both cases. In the case of the terbium silicide bilayer, the two mentioned modes have a frequency of 465.8 ${\mathrm{cm}}^{-1}$ and 244.5 ${\mathrm{cm}}^{-1}$, respectively, which matches quite well the frequencies of 467.6 ${\mathrm{cm}}^{-1}$ and 244.8 ${\mathrm{cm}}^{-1}$, respectively, predicted for the monolayer structure.

Other phonon modes are structure specific, instead. As an example, we mention a rigid vertical shift of the rare earth atoms. This mode of *A* symmetry is predicted at 31.1 ${\mathrm{cm}}^{-1}$ for the ${\mathrm{TbSi}}_{2}$ monolayer structure. In the case of the ${\mathrm{Tb}}_{3}$${\mathrm{Si}}_{5}$ bilayer reconstruction, there are two Tb layers (${\mathrm{RE}}_{1}$ and ${\mathrm{RE}}_{2}$ in Figure 4) instead of a single RE layer. This results in a much higher effective mass and, thus, in a much lower frequency of 20.1 ${\mathrm{cm}}^{-1}$. These structure specific modes can be in principle the key for a non-destructive, spectroscopic identification of the grown silicide structure. This is true in particular in low frequency region, where the low mode density and the absence of bulk resonances allow an unambiguous mode identification.

Among the bilayer specific modes, a couple of modes are strongly localized on the flat silicene-like layer ${\mathrm{Si}}_{3}$, shown in Figure 4b. In the case of terbium silicide, they are calculated at 402.4 ${\mathrm{cm}}^{-1}$ and 355.9 ${\mathrm{cm}}^{-1}$ (atomic displacements within the ${\mathrm{Si}}_{3}$ plane); 317.1 ${\mathrm{cm}}^{-1}$ (rotary mode within the ${\mathrm{Si}}_{3}$ layer); 265.7 ${\mathrm{cm}}^{-1}$, 264.2 ${\mathrm{cm}}^{-1}$, and 263.2 ${\mathrm{cm}}^{-1}$ (vertical displacements of the ${\mathrm{Si}}_{3}$ atoms); and 272.2 ${\mathrm{cm}}^{-1}$ and 193.5 ${\mathrm{cm}}^{-1}$ (rigid lateral translation and breathing mode, respectively). In homogeneous samples with a long-range silicide bilayer of ($\sqrt{3}\times \sqrt{3}$) periodicity, the observation of these modes should prove the existence of non buckled silicene-like layers, which have been controversially discussed in the literature [64].

**Figure 7:**
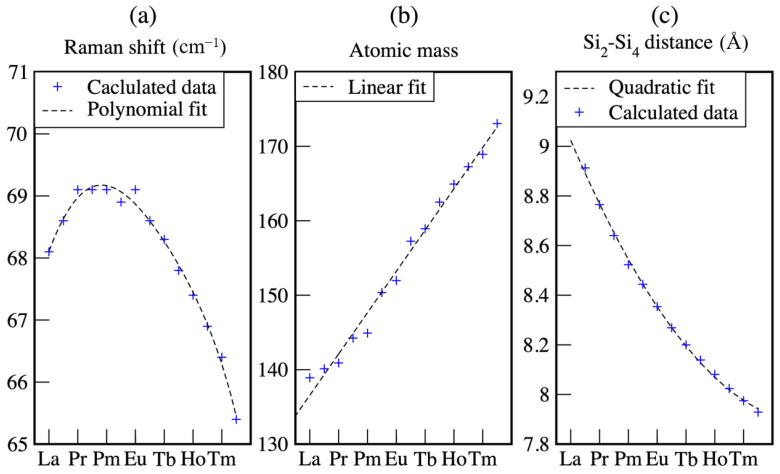
(**a**) Raman shifts of surface localized mode of the ${\mathrm{RE}}_{3}$${\mathrm{Si}}_{5}$-silicide bilayers with ($\sqrt{3}\times \sqrt{3}$)-periodicity calculated within DFT-PBEsol. (**b**) Atomic masses in the lanthanide series. (**c**) Silicide height as quantified by the ${\mathrm{Si}}_{2}$-${\mathrm{Si}}_{4}$ vertical distance. All quantities are shown as a function of the rare earth atomic number.

As in the case of the monolayer structure, most of the modes localized at the rare earth silicide bilayer show a moderate dependence on the rare earth. Most of the modes show a linear dependence, however, a more complex dependence is calculated for modes affecting the RE-Si distance. These modes show a slight frequency increase within the rare earth series and then a subsequent frequency decrease. An example is given by the Raman shift of the mode shown in Figure 7a. This mode is a lateral shearing of the two RE layers, which modifies the ${\mathrm{RE}}_{1}$-${\mathrm{Si}}_{3}$ and ${\mathrm{Si}}_{3}$-${\mathrm{RE}}_{2}$ bond lengths.

This behavior can be understood considering the factors determining the mode frequency. Considering the crystal lattice as a spring mass-point model, the squared mode frequency is directly proportional to the elastic constant of the spring and inversely proportional to the mass of the particle. The atomic mass of the rare earths grows roughly linearly with the atomic number, as shown in Figure 7b. Since we only consider trivalent rare earths, the RE-Si bonds is supposed to be of similar nature for all the considered lanthanides, and to depend only on the bond length. The latter however, does not decrease linearly with the atomic number, but decreases quickly for the light lanthanides and slowly for the heavy ones, as shown by the quadratic fit in Figure 7c. Thus, the mode frequency slightly grows due to the reduced bond lengths at the beginning of the lanthanide series. When the bond lengths reduction becomes less pronounced, the effect due to the atomic mass prevails, and the mode frequency decreases.

## 4. Discussion

The Raman signatures measured for terbium silicide layers grown on 4°-offcut Si(111) surfaces can be understood on the basis of the phonon modes calculated for the stoichiometric silicide monolayer of (1 × 1) periodicity grown on the Si(111) substrate. A displacement pattern can be unambiguously assigned to the measured spectral features. Indeed, both surface localized modes and activated bulk resonances are detected, which are in good agreement with the calculated modes. On the one hand, this suggests that the structures formed on the 4°-offcut Si(111) surface can be considered, in first approximation, as stoichiometric silicide monolayers. On the other hand, the good agreement between theory and calculations provides a strong argument for the validity of the silicide model we adopt to describe the monolayer structure.

Calculations extended to the monolayer structure of other rare-earth silicides demonstrate the dependence of the Raman shift on the rare-earth atomic-number. Roughly linear dependences are found, which correlate with the structural properties as determined by the lanthanide contraction.

Calculations performed on the silicide bilayer structure with ($\sqrt{3}\times \sqrt{3}$) periodicity, which models a higher rare-earth coverage, reveal the presence of structure specific modes not appearing in the monolayer termination. These modes have a complex dependence on the rare earth atomic number, which can be explained by the combined effect of bond lengths and atomic masses. The presence of Raman active, structure specific modes can be exploited for the non-destructive identification of the grown structure via Raman spectroscopy, provided homogeneous silicide layers can be grown.

According to our calculations, different modes of *A* and *E* symmetry are localized on the flat silicene-like layer separating the two silicide layers of the bilayer structure with ($\sqrt{3}\times \sqrt{3}$) periodicity. The experimental observation of these modes in future investigations might provide an argument for the existence of non-buckled silicene-like layers, which have been controversially discussed in the literature.

## Figures and Tables

**Table 1:** Measured Raman frequencies (in ${\mathrm{cm}}^{-1}$) of the Tb-reconstruction grown on 4°-offcut Si(111) substrate.

$4^{{}^{\circ}}$-Offcut z(yy)-z	$4^{{}^{\circ}}$-Offcut z(yx)-z	Symm.
41.5 ± 1.2	41.5 ± 1.3	*A*
92.0 ± 2		*A*
104.3 ± 1.1	103.9 ± 0.7	*E*
121.0 ± 0.7	121.9 ± 0.6	*E*
156.8 ± 1.2	154.6 ± 1.2	*E*
198.8 ± 0.8	197.9 ± 1.4	*A*
229.8 ± 0.9	229.0 ± 0.7	*A*
346.4 ± 0.8	345.5 ± 1.3	*A*
449.1 ± 1.2	448.7 ± 1.1	*A*

**Table 2:** Atomic layer distances (in Å) as calculated by DFT-PBEsol for the silicide monolayer structure with (1 × 1) periodicity of different rare earths. Labels as defined in Figure 4.

RE	${\mathrm{Si}}_{1}$-${\mathrm{Si}}_{2}$	${\mathrm{Si}}_{2}$-R	R-${\mathrm{Si}}_{3}$	${\mathrm{Si}}_{3}$-${\mathrm{Si}}_{4}$	${\mathrm{Si}}_{2}$-${\mathrm{Si}}_{3}$
La	0.711	2.072	2.247	0.887	4.319
Ce	0.706	2.055	2.221	0.885	4.276
Pr	0.719	2.017	2.175	0.889	4.191
Nd	0.732	1.981	2.139	0.894	4.120
Pm	0.746	1.946	2.105	0.899	4.051
Sm	0.757	1.922	2.081	0.902	4.003
Eu	0.762	1.903	2.052	0.904	3.955
Gd	0.774	1.878	2.026	0.908	3.903
Tb	0.784	1.857	2.001	0.911	3.862
Dy	0.793	1.839	1.988	0.914	3.827
Ho	0.795	1.829	1.969	0.915	3.798
Er	0.803	1.812	1.954	0.918	3.766
Tm	0.806	1.802	1.939	0.919	3.740
Yb	0.813	1.789	1.925	0.921	3.715

**Table 3:** Raman frequencies (in ${\mathrm{cm}}^{-1}$) calculated for the terbium silicide monolayer with (1 × 1) periodicity within DFT-PBEsol. Surface localization, mode symmetry, Raman activity, and possible assignment to the measured Raman signatures are reported.

Freq.	Symm.	Local.	Surf-Bulk	Active	Assign.
502.9	*E*	1.7%	Res.	Y	Outside window
500.0	*A*	24.7%	Res.	Y	Outside window
494.4	*E*	2.3%	Res.	N	Outside window
488.5	*E*	1.1%	Res.	Y	Outside window
485.0	*A*	23.8%	Res.	Y	Outside window
467.6	*E*	92.7%	Surf.	Y	Outside window
451.1	*A*	20.9%	Res.	Y	449.1
414.3	*E*	97.0%	Surf.	N	Raman silent
388.1	*A*	18.1%	Res.	Y	Not localized
333.5	*A*	29.4%	Res.	Y	346.4
264.2	*A*	34.5%	Res.	Y	Not localized
244.7	*A*	82.5%	Surf.	Y	229.8
199.1	*A*	51.1%	Surf.	Y	198.8
166.8	*A*	70.5%	Surf.	Y	
151.6	*E*	97.7%	Surf.	Y	156.8
117.8	*E*	84.9%	Surf.	Y	121.0
110.8	*A*	36.4%	Surf.	Y	92.0
98.8	*E*	8.6%	Res.	N	Raman silent
82.3	*E*	19.2%	Res.	Y	104.3
51.5	*E*	25.3%	Res.	N	Raman silent
31.1	*A*	70.5%	Surf.	Y	41.5
15.5	*E*	65.2%	Surf.	Y	Outside window

**Table 4:** Atomic layer distances (in Å) calculated by DFT-PBEsol for the silicide bilayer structure with ($\sqrt{3}\times \sqrt{3}$) periodicity for different rare earths. Labels as defined in Figure 4.

RE	${\mathrm{Si}}_{1}$-${\mathrm{Si}}_{2}$	${\mathrm{Si}}_{2}$-$R_{1}$	$R_{1}$-${\mathrm{Si}}_{3}$	${\mathrm{Si}}_{3}$-$R_{2}$	$R_{2}$-${\mathrm{Si}}_{4}$	${\mathrm{Si}}_{4}$-${\mathrm{Si}}_{5}$	${\mathrm{Si}}_{2}$-${\mathrm{Si}}_{3}$	$R_{1}$-$R_{2}$	${\mathrm{Si}}_{2}$-${\mathrm{Si}}_{4}$
La	0.709	2.214	2.271	2.217	2.282	0.838	4.485	4.487	8.983
Ce	0.710	2.190	2.245	2.196	2.280	0.840	4.436	4.441	8.913
Pr	0.720	2.150	2.209	2.159	2.246	0.841	4.360	4.369	8.765
Nd	0.728	2.116	2.179	2.128	2.217	0.843	4.295	4.307	8.640
Pm	0.737	2.084	2.150	2.098	2.192	0.845	4.234	4.248	8.523
Sm	0.745	2.062	2.130	2.076	2.176	0.847	4.192	4.206	8.444
Eu	0.752	2.038	2.107	2.053	2.155	0.849	4.145	4.160	8.354
Gd	0.760	2.015	2.084	2.030	2.139	0.852	4.100	4.114	8.269
Tb	0.767	1.997	2.066	2.011	2.126	0.855	4.063	4.077	8.200
Dy	0.774	1.981	2.050	1.993	2.115	0.858	4.030	4.043	8.139
Ho	0.780	1.965	2.034	1.977	2.105	0.861	3.999	4.011	8.081
Er	0.787	1.949	2.020	1.961	2.095	0.864	3.969	3.980	8.024
Tm	0.793	1.935	2.007	1.947	2.087	0.868	3.942	3.954	7.975
Yb	0.799	1.921	1.994	1.933	2.080	0.871	3.916	3.927	7.929

**Figure A1:**
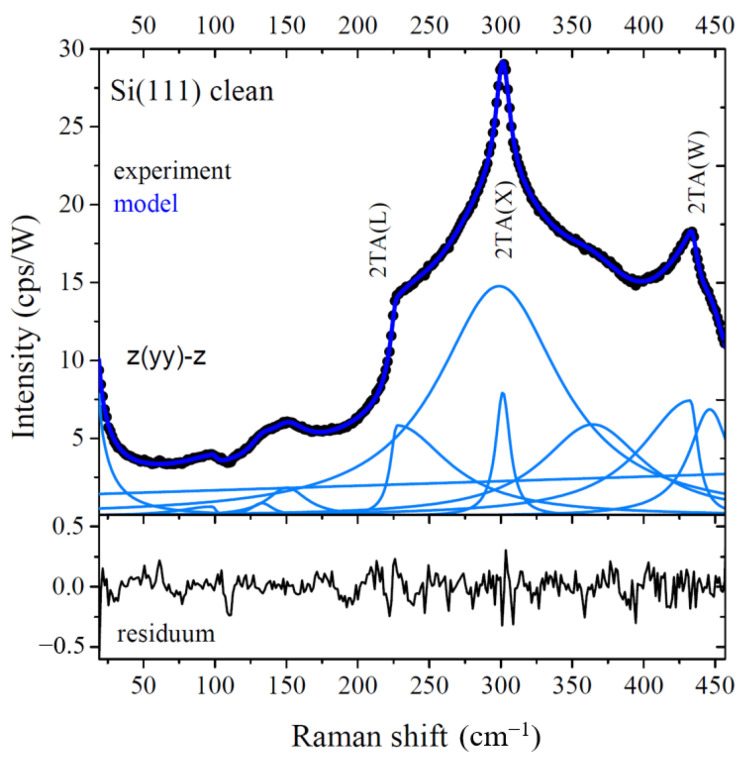
Parametrization of the Raman spectrum of clean Si(111). The critical points *L*, *X*, *W* of the 2TA band of silicon are indicated [65]. The experimental spectrum (black dots) is well approximated by the model (blue curve), as shown by the low residuum (lower panel).

## Data Availability

Data shown in this paper are available from the authors upon reasonable request.

## Appendix A. Separation of Bulk and Surface Contributions in Raman Spectra

The measured Raman spectra contain bulk and surface contributions. The surface Raman spectra shown in Figure 2 in the main text are obtained by subtracting a parametrization of the bulk contributions from the measured spectra. The parametrization consists in modeling the Raman spectra with a combination of different functions, as shown in Figure A1. A Lorentz function centered at 0 ${\mathrm{cm}}^{-1}$ models the laser line, a linear function models setup alignment fluctuations and secondary effects, and asymmetric Lorentz functions model the Si phonon density of states. This parametrization is not intended to be a rigorous model of the Raman spectrum of Si, rather a practical method to separate surface from bulk contributions.

A comparison between measured and parametrized spectra of the clean Si(111) surface is shown in Figure A2. Surface spectra could be extracted by direct subtraction of the spectra of the reconstructed and clean surface shown in Figure A1, a method already applied in past works [52,66]. The disadvantages of this method are the increase in the noise level in the difference spectra and the possible presence of artifacts due to the limited reproducibility of the experimental conditions (alignment, scattering, etc.). The parametrization method is proposed as method to overcome these disadvantages by adaption to the surface spectra, as shown in Figure A2.

## Appendix B. List of Calculated Surface Localized Modes

Here we show the full list of surface localized phonon modes calculated for the monolayer and for the bilayer structure of the different rare earth silicides.

**Table A1:** Raman frequencies (in ${\mathrm{cm}}^{-1}$) calculated for the silicide monolayer with (1 × 1) periodicity for different rare earths within DFT-PBEsol.

Sym.	La	Ce	Pr	Nd	Pm	Sm	Eu	Gd	Tb	Dy	Ho	Er	Tb	Yb
*E*	502.6	502.6	502.6	502.7	502.8	502.7	502.8	502.9	502.9	502.8	502.7	502.8	502.8	502.9
*A*	494.9	495.3	496.9	498.4	498.8	497.2	497.4	499.4	500.0	498.7	498.5	500.2	497.9	500.2
*E*	494.1	494.2	494.2	494.3	494.3	494.2	494.4	494.5	494.4	494.3	494.5	494.5	494.5	494.5
*E*	488.4	488.2	488.3	488.3	488.4	488.3	488.5	488.5	488.5	488.4	488.5	488.6	488.5	488.5
*A*	479.2	479.1	481.5	483.0	483.5	482.8	483.4	484.6	485.0	484.3	484.3	485.2	484.6	485.5
*E*	489.2	489.0	484.6	481.8	478.8	475.8	473.5	470.4	467.6	464.6	462.1	459.4	458.6	457.1
*A*	449.0	448.6	449.1	449.8	450.1	450.0	450.5	450.8	451.1	450.8	451.0	451.2	451.1	451.3
*E*	426.3	426.2	421.8	420.7	420.2	419.6	416.7	415.7	414.3	413.4	417.5	418.6	411.7	410.3
*A*	387.4	387.3	387.4	387.6	387.8	387.7	387.8	388.0	388.1	388.0	387.8	388.0	388.0	388.1
*A*	334.1	333.4	333.2	333.4	333.6	333.4	333.3	333.5	333.5	333.2	332.9	333.4	333.1	333.2
*A*	264.0	264.1	263.6	263.6	263.9	263.8	264.1	264.1	264.2	263.7	263.4	264.2	263.4	264.1
*A*	226.2	223.4	229.7	236.4	238.6	233.6	234.1	242.0	244.7	235.5	232.1	238.3	232.6	245.0
*A*	188.3	191.2	193.2	196.4	196.9	194.7	196.3	198.1	199.1	197.0	197.0	197.6	198.8	198.7
*A*	160.3	159.4	163.2	165.3	166.0	163.9	164.4	165.9	166.8	165.3	165.1	164.1	165.9	164.8
*E*	147.7	147.1	148.5	150.1	152.0	151.5	151.5	151.5	151.6	150.6	148.9	148.8	147.7	147.8
*E*	112.0	113.3	114.6	115.5	116.4	116.8	117.3	117.7	117.8	118.0	117.6	117.5	117.6	117.2
*A*	103.0	104.5	106.6	107.7	108.6	108.2	109.5	110.6	110.8	110.3	111.2	112.4	111.5	112.7
*E*	98.9	98.5	98.6	98.7	98.8	98.9	98.7	98.7	98.8	98.8	98.9	99.0	98.8	98.8
*E*	81.6	81.7	82.1	82.2	82.3	82.4	82.3	82.5	82.3	82.5	82.5	82.5	82.4	82.4
*E*	51.6	51.5	51.6	51.7	51.8	51.8	51.7	51.8	51.5	51.5	51.8	51.7	51.6	51.3
*A*	32.9	32.9	31.6	29.7	30.8	31.5	31.7	31.0	31.1	30.9	31.1	31.5	30.3	30.8
*E*	16.3	16.3	16.3	16.2	16.3	15.9	15.9	15.7	15.5	15.5	15.6	15.4	15.6	15.7

**Table A2:** Raman frequencies (in ${\mathrm{cm}}^{-1}$) calculated for the silicide bilayer with ($\sqrt{3}\times \sqrt{3}$) periodicity for different rare earths within DFT-PBEsol. Only phonon modes localized by more than 50% within the atomic layers ${\mathrm{Si}}_{1}$-${\mathrm{Si}}_{5}$ are shown. The localization is calculated in the case of terbium silicide.

Sym.	La	Ce	Pr	Nd	Pm	Sm	Eu	Gd	Tb	Dy	Ho	Er	Tb	Yb
*E*	486.2	486.4	483.4	480.7	477.9	475.7	473.3	469.2	465.8	463.9	463.8	463.8	462.2	458.8
*E*	459.7	460.7	457.8	455.1	452.2	450.1	448.1	445.3	443.4	441.4	439.5	437.5	436.1	434.0
*E*	448.0	446.5	444.9	443.6	442.1	440.7	439.4	437.7	436.3	434.8	433.4	431.5	427.5	425.6
*E*	428.1	427.5	426.3	425.2	423.8	422.3	420.9	418.9	417.2	415.5	413.9	412.7	411.8	410.3
*E*	416.5	416.2	414.5	412.7	410.7	408.5	407.3	404.5	402.4	400.3	398.3	396.7	395.1	392.2
*E*	374.0	376.0	374.8	373.6	372.2	371.0	369.9	368.5	367.2	365.9	364.5	363.2	361.9	360.5
*A*	381.1	379.4	377.7	375.9	374.0	371.9	370.4	367.7	365.5	363.3	361.5	360.0	358.8	357.0
*A*	378.6	377.6	375.8	374.1	372.1	370.2	368.5	366.0	363.8	361.6	359.2	357.7	355.7	353.4
*E*	367.0	366.7	365.1	363.6	362.1	360.3	359.5	357.5	355.9	354.4	352.7	351.7	350.9	349.1
*E*	351.0	352.8	352.7	352.7	352.3	352.1	351.8	351.0	350.4	349.9	349.3	348.6	347.9	347.0
*A*	319.8	323.2	322.7	322.1	321.2	320.1	320.0	318.3	317.1	315.8	314.4	313.5	312.6	310.7
*A*	262.7	264.6	266.6	267.7	267.3	269.5	271.8	271.6	272.2	272.8	273.7	275.2	276.1	276.2
*E*	250.1	256.8	258.6	260.2	261.6	262.9	264.7	265.1	265.7	266.3	267.2	268.9	269.3	268.7
*A*	240.7	249.5	252.3	254.1	256.8	257.8	261.0	262.7	264.2	265.6	267.2	269.5	271.1	271.2
*A*	240.1	245.0	248.2	250.8	254.0	255.6	259.4	261.4	263.2	264.8	266.3	268.0	268.7	268.9
*A*	231.6	239.1	240.4	241.4	242.4	243.1	244.1	244.4	244.5	244.8	245.1	244.8	243.5	244.7
*A*	227.9	226.6	226.8	226.9	226.9	226.9	227.1	226.7	226.5	226.3	226.0	225.8	225.6	225.0
*A*	222.0	221.4	221.2	220.9	220.6	220.2	219.9	219.2	218.7	218.2	218.0	217.4	216.9	216.3
*E*	208.0	216.1	219.0	221.1	223.1	224.0	226.3	226.6	227.2	227.6	228.1	228.8	229.4	228.4
*E*	201.6	201.2	201.1	201.0	200.8	200.5	200.4	200.1	199.8	199.5	199.4	199.2	198.0	197.8
*E*	201.4	201.1	201.5	201.7	201.7	201.4	201.5	200.9	200.4	199.9	199.1	198.8	198.4	197.3
*A*	197.8	199.0	198.3	197.7	196.8	196.1	195.9	194.4	193.5	192.9	192.0	191.0	190.2	190.0
*E*	168.8	175.2	176.0	176.6	177.1	177.0	178.2	177.5	177.1	177.1	176.7	177.3	177.0	176.4
*E*	156.8	156.2	156.3	155.8	155.3	154.3	154.4	153.0	152.4	151.7	151.0	150.5	149.8	148.3
*A*	144.9	149.9	151.7	152.7	153.6	154.3	155.1	152.2	150.9	150.7	152.0	154.4	154.3	152.4
*A*	136.0	136.7	135.3	132.9	130.6	128.0	126.6	123.2	121.4	119.0	117.0	117.1	116.5	115.6
*E*	135.2	131.3	130.1	129.1	128.3	127.4	127.4	125.9	125.0	123.9	123.2	122.9	122.3	120.7
*E*	125.3	125.5	126.3	125.9	125.0	123.8	122.7	121.2	120.1	119.1	118.5	118.1	117.6	116.6
*A*	124.6	126.7	125.9	124.0	122.3	120.2	120.2	118.5	118.3	117.3	117.0	115.4	113.6	111.4
*A*	120.3	122.7	122.6	121.8	121.2	120.0	118.9	115.9	114.2	111.8	109.8	107.7	105.8	103.3
*E*	118.2	118.3	119.1	119.3	119.4	119.0	119.1	118.2	117.4	116.6	115.7	114.8	114.0	113.1
*E*	114.9	114.3	114.9	114.4	113.8	112.6	112.5	110.4	109.4	108.0	107.2	106.6	105.8	103.5
*A*	104.6	104.8	105.0	104.6	104.3	103.9	104.2	102.9	102.5	101.8	101.6	100.7	99.4	98.1
*E*	108.8	110.8	109.8	108.0	106.4	104.3	103.1	100.1	98.4	96.2	94.6	93.2	92.4	90.8
*E*	98.8	100.5	99.6	98.2	97.1	96.0	96.4	94.9	94.7	93.7	92.9	91.5	89.9	87.4
*A*	94.4	101.3	100.0	97.9	95.8	94.2	93.5	94.2	94.2	94.2	94.4	94.6	93.9	93.9
*E*	93.5	94.1	95.3	95.4	95.3	94.2	93.3	91.0	89.4	87.6	86.1	84.9	83.4	81.7
*A*	86.6	93.7	94.6	94.6	94.9	93.5	91.4	87.3	84.5	82.2	80.8	79.8	78.2	76.7
*A*	77.6	77.6	77.9	77.9	77.8	77.7	77.7	77.6	77.4	77.2	77.1	77.3	76.3	74.9
*E*	68.1	68.6	69.1	69.1	69.1	68.9	69.1	68.6	68.3	67.8	67.4	66.9	66.4	65.4
*A*	53.5	64.2	64.2	64.3	64.4	64.2	64.8	63.9	64.5	63.8	63.7	64.0	62.8	61.8
*A*	21.2	21.1	20.9	20.7	20.4	20.5	20.5	20.2	20.1	19.8	19.2	18.8	19.3	19.1
*E*	11.7	11.6	11.5	11.4	11.4	11.4	11.5	11.3	11.2	11.2	11.1	11.0	10.7	10.5
